# Unveiling the Dynamics of Thermal Characteristics Related to LULC Changes via ANN

**DOI:** 10.3390/s23157013

**Published:** 2023-08-07

**Authors:** Yasir Hassan Khachoo, Matteo Cutugno, Umberto Robustelli, Giovanni Pugliano

**Affiliations:** 1 Department of Engineering, University of Naples Parthenope, 80143 Naples, Italy; yasirhassan.khachoo001@studenti.uniparthenope.it (Y.H.K.); umberto.robustelli@uniparthenope.it (U.R.); 2 University of Benevento Giustino Fortunato, 82100 Benevento, Italy; 3 Department of Civil, Architectural and Environmental Engineering, University of Naples Federico II, 80125 Naples, Italy; giovanni.pugliano@unina.it

**Keywords:** Urban Thermal Field Variance Index (UTFVI), temporal analysis, thermal discomfort, Province of Naples, prediction

## Abstract

Continuous and unplanned urbanization, combined with negative alterations in land use land cover (LULC), leads to a deterioration of the urban thermal environment and results in various adverse ecological effects. The changes in LULC and thermal characteristics have significant implications for the economy, climate patterns, and environmental sustainability. This study focuses on the Province of Naples in Italy, examining LULC changes and the Urban Thermal Field Variance Index (UTFVI) from 1990 to 2022, predicting their distributions for 2030. The main objectives of this research are the investigation of the future seasonal thermal characteristics of the study area by characterizing land surface temperature (LST) through the UTFVI and analyzing LULC dynamics along with their correlation. To achieve this, Landsat 4-5 Thematic Mapper (TM) and Landsat 9 Operational Land Imager (OLI) imagery were utilized. LULC classification was performed using a supervised satellite image classification system, and the predictions were carried out using the cellular automata-artificial neural network (CA-ANN) algorithm. LST was calculated using the radiative transfer equation (RTE), and the same CA-ANN algorithm was employed to predict UTFVI for 2030. To investigate the multi-temporal correlation between LULC and UTFVI, a cross-tabulation technique was employed. The study’s findings indicate that between 2022 and 2030, there will be a 9.4% increase in built-up and bare-land areas at the expense of the vegetation class. The strongest UTFVI zone during summer is predicted to remain stable from 2022 to 2030, while winter UTFVI shows substantial fluctuations with a 4.62% decrease in the none UTFVI zone and a corresponding increase in the strongest UTFVI zone for the same period. The results of this study reveal a concerning trend of outward expansion in the built-up area of the Province of Naples, with central northern regions experiencing the highest growth rate, predominantly at the expense of vegetation cover. These predictions emphasize the urgent need for proactive measures to preserve and protect the diminishing vegetation cover, maintaining ecological balance, combating the urban heat island effect, and safeguarding biodiversity in the province.

## 1. Introduction

Cities play a crucial role in a country’s development, as they facilitate economic growth, employment opportunities, infrastructure development, innovation, and cultural diversity. They act as central hubs for transportation, telecommunications, energy, and water services. The increasing effects of global warming along with unplanned city infrastructure, including poorly designed and congested road networks, disorganized building layouts, and inadequate green spaces, contribute to environmental degradation, disasters (like building collapses, floods, and landslides), social inequality, and economic inefficiencies. Continuous unplanned urbanization and negative modifications in land use land cover (LULC) lead to a deteriorating urban thermal environment, resulting in various adverse ecological effects, such as increased air pollution [1] and higher energy demand [2]. The intensity of thermal stress negatively affects air quality, diurnal temperature range, wind patterns, vegetation phenology, humidity, water consumption, living comfort, indirect economic losses, and mortality rates [1,3,4,5]. Furthermore, it reduces a city’s ability to sustain itself and increases vulnerability to regional and global climate change. As a result, environmentally beneficial LULC types such as forest cover and natural water supplies are continuously diminishing, exacerbating the effects of urban thermal characteristics [6].

In recent years, there has been increasing research focusing on thermal characteristics, their impacts, and contributing factors, particularly in mega-cities. Liu and Weng [7] investigated the scaling-up effect on the relationships between LULC and land surface temperature (LST), determining that a 30 m spatial resolution is enough for exploring LULC and LST patterns. AlDousari et al. [8] studied the effects of LULC changes (LULCC) on thermal characteristics in Kuwait using machine learning algorithms. Italy, in particular, experienced significant landscape transformations [9], necessitating an evaluation of changes in the thermal comfort of its cities in relation to historical LULCC.

Remote sensing techniques offer advanced support for monitoring spatio-temporal LULCC, ecological conditions, and thermal patterns at local, regional, and global scales [10]. Satellite missions, such as Landsat and Sentinel, coupled with improvements in the accuracy of global navigation satellite system (GNSS) techniques [11,12,13,14], provide freely available remotely sensed images that can be used for obtaining LULC and LST [15,16]. These are key factors for understanding processes, such as land–atmosphere interactions, climate change, and the urban heat island effect. Over the past years, several techniques for LULC classification, including supervised [17], unsupervised [18], and object based [19,20], have been employed. There are different approaches and algorithms for LST calculation using Landsat imagery. Du et al. [21] developed a practical split-window algorithm to estimate LST from thermal infrared sensor (TIRS) aboard Landsat 8, obtaining LST with an accuracy better than 1.0 K; Maithani et al. [22] retrieved LST from Landsat thermal datasets using a single-channel algorithm for the Dehradun planning area situated in Uttarakhand (India); Yu et al. [23] compared three different approaches for LST inversion from TIRS, including the radiative transfer equation-based method, the split-window algorithm, and the single-channel method. Their findings indicated that the LST obtained from the radiative transfer equation-based method, using Landsat band 10, has the highest accuracy with RMSE lower than 1.0 K, while the split-window algorithm has moderate accuracy and the single-channel method has the lowest accuracy. Once calculated, various methods can be used to characterize LST. Kafy et al. [24] evaluated the LST of Sylhet city, Bangladesh, with Landsat images from 1995 to 2020, by employing the Urban Thermal Field Variance Index (UTFVI). Furthermore, Renard et al. [25] demonstrated that the UTFVI is an efficient index to quantitatively analyze the urban heat island effect. This effect was also assessed by Du et al. [26], which classified LST by exploiting the standard deviation method in a typical mega-city like Shangai (China). For what concerns the prediction of future scenarios, various machine learning algorithms have already been developed by researchers, among which include artificial neural networks (ANNs) based on cellular automata (CA), random forest [27], and support vector regression [28]. Sajan et al. [29] assessed the past, present and future changes in LULC of the Muzaffarpur district (India) using CA-ANN algorithms. They used Landsat data for various years demonstrating the ability of the model to forecast future events and comprehend spatio-temporal LULC dynamics. For what concerns the relevant studies about the thermal characteristics regarding the Southern Italy area, few works in the literature can be found. Oliveira et al. [30] predicted the LST patterns for the province of Naples by means of a random forest (RF) approach, accurately predicting the LST. Their findings indicate that the maximum net heat flux occurs approximately during the solar noon, with the diurnal curve following the solar radiation cycle, and negative heat flux values are observed during the night. They also discovered that no significant differences exist between the urban and rural sites. The sensible, storage, and latent heat flux components show contrasting profiles since in the rural site, the evapotranspiration surface heat loss accounts for more than half of the available net flux energy, whereas the sensible heat flux component does not reach such values, in the same period. In the urban site, these proportions are reversed. Moreover, Guha et al. [31] utilized Landsat-8 data to analyze surface urban heat island (SUHI) patterns and UTFVI maps in Naples and Florence, Italy. Their findings demonstrated that over 75% of SUHIs originated in areas classified as bare land and built up, which were also identified as ecologically stressed zones. While Oliveira et al. [30] conducted a study in the same area limited to a particular heat wave event, the aim of this paper is to provide a prediction for future LULC dynamics, thermal characteristics, and their correlation. Therefore, the results of this research will enable urban planners, policymakers, and local governments to promote environmentally friendly, inclusive, and sustainable urban development. This will be achieved by strategically modifying and replacing the distribution of LULC based on both current and future conditions. In particular, the characterization of the specific responses of future seasonal thermal characteristics in response to LULCC can reveal valuable information. The study focuses on the Province of Naples in Italy, examining LULCC from 1990 to 2022 and predicting future LULC patterns and UTFVI distributions for 2030. The main objective of this study is the investigation of the future seasonal thermal characteristics of the Province of Naples by characterization of LST retrieved from the thermal bands of the Landsat satellite imagery analyzing the future LULC dynamics. This was performed by using a supervised satellite image classification system and CA-ANN prediction algorithm. Furthermore, an analysis of LULC and UTFVI spatial distributions was carried out. Indeed, the findings of this study can be used to inform urban-planning decisions, assess areas under heat stress, and support climate change studies with respect to local temperature dynamics and LULC changes in the Province of Naples.

The paper is structured as follows: Section 2 defines the materials and the methods employed. Section 3 presents and discuss the outcomes. Lastly, Section 4 draws some conclusions and defines the future lines of research.

## 2. Materials and Methods

### 2.1. Study Area (ROI)

**Figure 1:**
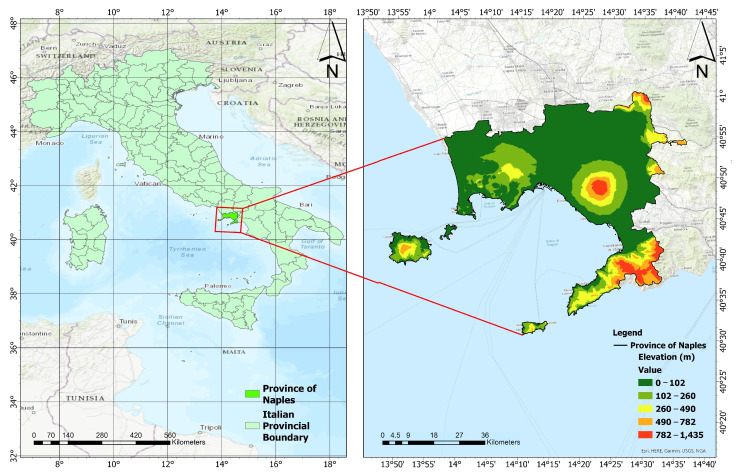
Location of the ROI: (**left panel**) shows the Italian peninsula with provincial boundaries, whereas (**right panel**) is a zoomed-in version focused on the Province of Naples reporting its DEM.

The Province of Naples serves as the administrative hub for the Campania region and is the third-largest metropolitan area in Italy. The selection of the Province of Naples for this study is based on its status as one of the most densely populated area in Italy and the lack of previous investigations into its thermal features in relation to LULCCs. The left panel of Figure 1 shows the Italian peninsula with provincial boundaries. Geographically, the Province of Naples is situated in Southern Italy along the Tyrrhenian Sea coast. Its latitude ranges from 40°38${}^{\prime }$ N to 41°21${}^{\prime }$ N, and its longitude ranges from 13°55${}^{\prime }$ E to 14°44${}^{\prime }$ E. The right panel of Figure 1 depicts the Province of Naples and its digital elevation model (DEM). The province covers an approximate area of 1173.16 km${}^{2}$ and comprises 92 municipalities, with a total population of approximately 3.05 million. The city of Naples by itself accommodates nearly a million people within an area of approximately 117 km${}^{2}$. The province experiences a typical Mediterranean climate characterized by cool and damp winters and hot and dry summers. The average daily temperature ranges from 9 °C in January to 26 °C in August, and annual rainfall exceeds 1000 millimeters. The wettest season occurs during autumn, particularly in the months of October and November, followed by the winter months.

### 2.2. Methodology

**Figure 2:**
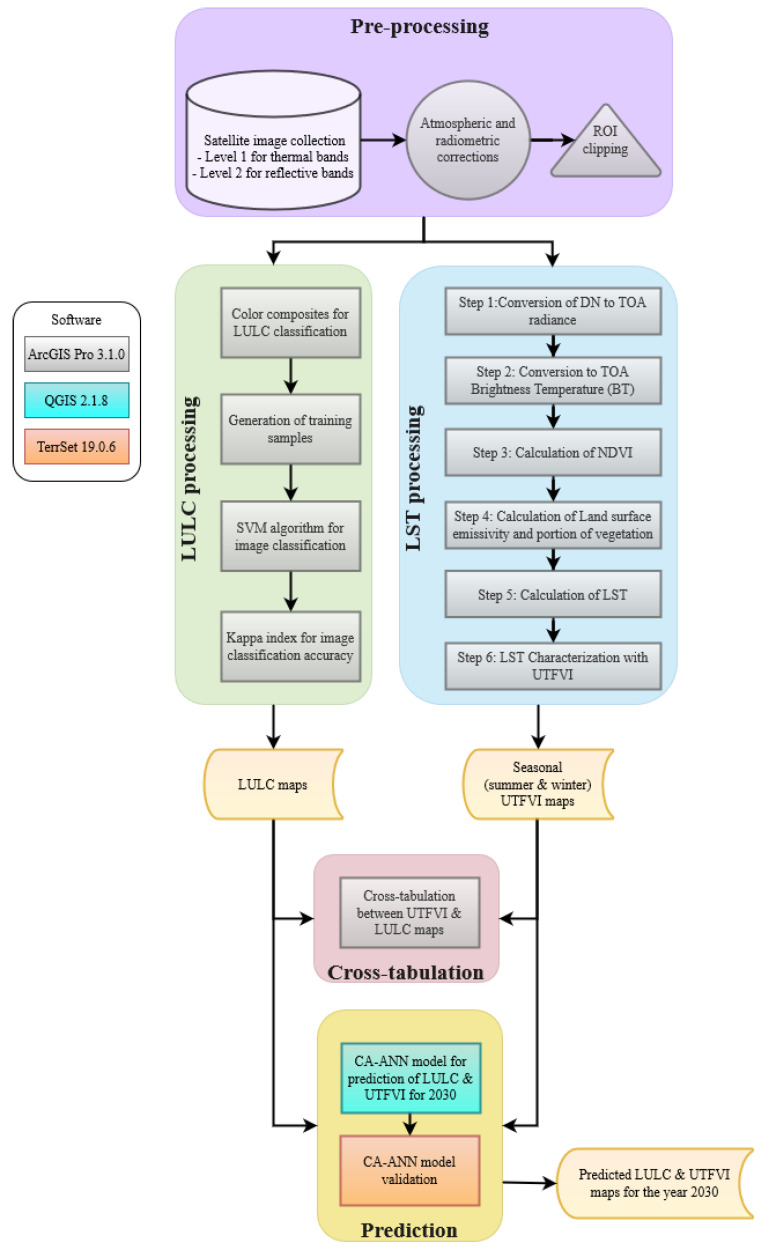
Flow chart of the methodology utilized. The violet background box encloses the pre-processing steps. The left leg, contained in a light green background box depicts the LULC processing. The right leg, contained in a light blue background box, shows the LST processing. The background box colored in pink indicates the cross-tabulation stage and, lastly, the yellow background box shows the prediction stage. The inner boxes colored in grey are performed with ArcGIS Pro software, the one colored in blue is carried out in QGIS, and the one in orange is achieved with TerrSet software.

Figure 2 illustrates the methodology employed in this study. It encompasses several key steps. Firstly, the thermal bands of Level-1 satellite images collected were pre-processed for atmospheric and radiometric corrections. Then, LULC classification was achieved through the pixel-based supervised method [17] applied on Level-2 satellite images. This was chosen for its light computational requirements. LULC results were validated by means of the kappa index [32]. LST retrieval was conducted with the radiative transfer equation (RTE) approach. LST information can be obtained from the radiation emitted by the body of any structure via the inversion of Planck’s law. This approach was preferred due to its high accuracy for band 10 of Landsat 8-9 OLI as reported in [33] and its ability to retrieve LST from a single thermal band in contrast to the split-window algorithm, which needs two thermal bands. Furthermore, the mono-window algorithm depends on effective mean atmospheric temperature.

LST was characterized by using the UTFVI due to its efficient capability to assess the thermal characteristics of urban areas [24]. The UTFVI is commonly used by the scientific community to assess the thermal quality of urban areas. It can identify urban hot spots, and a cross tabulation between UTFVI and LULC can help in revealing valuable insights [25]. The UTFVI is divided into six levels, where each level represents a different thermal scenario. The six levels provided by the UTFVI correspond with the six specific ecological evaluation indices [34].Lastly, the prediction stage was achieved by means of the CA-ANN modeling approach [35] for its accurate performance in simulating future LULC changes by considering previous trends and interactions between LULC classes [24]. Employing predictive modeling to anticipate the effects of future LULC, LST, and UTFVI can serve as a valuable approach in identifying potential heat-prone areas. By doing so, it enables the implementation of essential measures to ensure a sustainable urban environment and effectively address heat-related challenges.

#### 2.2.1. Satellite Image Collection and Pre-Processing

The study utilized a total of eight Landsat satellite images, specifically Landsat Collections Level-1 products for thermal bands used in LST processing and Landsat Collections Level-2 products reflective bands for LULC processing. These images were captured at about 10-year intervals (1990, 2000, 2010, and 2022). The images were downloaded from the United States Geological Survey (USGS) Earth Explorer website [36], considering only the images, where the cloud cover was less than 10%. Particularly, for LST processing, for the year 1990, the study employs images from Landsat 4 (bands 3, 4, and 6), whereas for the years 2000 and 2010, the study employs images from Landsat 5 (bands 3, 4, and 6). Lastly, for the year 2022, the images considered belong to Landsat 9 (bands 4, 5, and 10), given the decommissioning of Landsat 4-5. The thermal bands of satellite images collected were corrected for atmospheric and radiometric distortions, according to the procedure detailed in [37,38]. For LULC processing, the study employs, for the years 1990, 2000, and 2010, bands 1 to 5 and 7 from Landsat 4-5, whereas for the year 2022, the images considered belong to Landsat 9 (bands 1 to 7). According to the USGS bulletin, published in 2010 [39], all thermal bands were resampled by USGS to a 30 m spatial resolution, given the difficulties encountered by commercial software to align them to the 30 m multispectral bands. The characteristics of the downloaded imagery, used for LST computation, can be found in Table 1. To explore the seasonal variations in UTFVI in relation to LULCCs, a set of four images was selected for the summer season (July–August) and another four for the winter season (December–January).

#### 2.2.2. LST Processing

##### Step 1: Conversion of DN to TOA Radiance

To retrieve LST, the RTE approach was utilized since it is not dependent on average temperature and air humidity. Furthermore, according to Yu et al. [23], the RTE-based approach has the highest accuracy for LST retrieval using Landsat band 10. For the purpose of collecting the temperature of the region of interest (ROI), over a specific time period, two different types of Landsat images were considered. The satellite images were then processed using the procedure in the handbook downloaded from USGS [37,38].

The following procedure was applied to thermal bands to obtain LST. Firstly, using the radiometric rescaling coefficients contained in the metadata file, included within the Level-1 product, the thermal band from the Landsat 4-5 TM was scaled to top of atmosphere (TOA) spectral radiance using the following equation:

(1) $$L_{\lambda }=M_{L}Q_{cal}+A_{L}$$

where the $M_{L}$ band-specific multiplicative rescaling factor has a value of $5.5375\times 10^{-2}$ for Landsat 4-5 TM and $3.8000\times 10^{-4}$ for Landsat 9 OLI; $A_{L}$ is the band-specific additive rescaling factor with a value of 1.18243 for Landsat 4-5 TM and 0.1000 for Landsat 9 OLI, both provided in the meta data file; $Q_{cal}$ is the calibrated and quantized standard product pixel values; and $Q_{i}$ is a correction factor with a value of 0.29.

##### Step 2: Conversion to TOA Brightness Temperature (BT)

Then, thermal band 6 from Landsat 4-5 TM and thermal band 10 from Landsat 9 OLI were converted to the TOA brightness temperature, using the following equation:

(2) $$BT=(\frac{K_{2}}{ln(\frac{K_{1}+L_{\lambda }}{L_{\lambda }})})-273.15$$

where BT is the TOA brightness temperature in degrees Celsius; $K_{1}$ and $K_{2}$ are the dimensionless band-specific thermal conversion constants from the metadata file; the thermal constant $K_{1}$ has values of 607.76 and 799.0284 for Landsat 4-5 TM and for Landsat 9 OLI, respectively; and the thermal constant $K_{2}$ has values of 1260.56 and 1329.2405 for Landsat 4-5 TM and for Landsat 9 OLI, respectively.

##### Step 3: Calculation of NDVI

It follows the calculation of the Normalized Difference Vegetation Index (NDVI) using the following formula:

(3) $$NDVI=\frac{\left(NIR-Red\right)}{\left(NIR+Red\right)}$$

where near infrared (NIR) is band 4 of Landsat 4-5 TM and band 5 of Landsat 9 OLI, and red is band 3 of Landsat 4-5 TM and band 4 of Landsat 9 OLI.

##### Step 4: Calculation of Land Surface Emissivity and Portion of Vegetation

The next step is the calculation of the land surface emissivity to identify the portion of vegetation. The land surface emissivity (ϵ) is a measure of the efficiency with which a land surface emits thermal radiation at a particular wavelength. The ϵ is calculated by the following formula:

(4) $$\epsilon =0.004P_{V}+0.986$$

where $P_{V}$ is the portion of vegetation, which is calculated as

(5) $$P_{V}={\left(\frac{\left(NDVI-NDVI_{min}\right)}{\left(NDVI_{max}-NDVI_{min}\right)}\right)}^{2}$$

where $NDVI_{min}$ and $NDVI_{max}$ are the minimum and maximum NDVI values of the image, respectively.

##### Step 5: Calculation of LST

After the identification of the portion of vegetation, the core calculation of the study, which is the calculation of LST, was performed. First, the value of ρ was calculated as

(6) $$\rho =(\frac{h*c}{\sigma })$$

where *h* is the Planck constant with a value of $6.626\times 10^{-34}$ Js, *c* is the speed of light with a value of $2.998\times 10^{8}\;{\mathrm{ms}}^{-1}$, and σ is the Boltzmann constant with a value of $1.380649\times 10^{-23}\;{\mathrm{JK}}^{-1}$. Then, LST is calculated using the following formula:

(7) $$LST=(\frac{BT}{1+\left(\frac{\lambda BT}{\rho }\right)ln\left(\epsilon \right)})$$

where λ is the wavelength of the emitted radiance. The value of λ for the Landsat 4-5 TM thermal band (band 6) is 11.46μm and the value of λ for Landsat 9 OLI (band 10) is 10.89μm.

##### Step 6: LST Characterization with UTFVI

The last step is the characterization of LST based on UTFVI. The seasonal UTFVI was calculated to evaluate the thermal properties of the ROI. UTFVI was calculated according to Equation (8) using the LST values obtained in Equation (7), as follows:

(8) $$UTFVI=(\frac{LST-LST_{mean}}{LST_{mean}})$$

To determine the relationship between UTFVI and LULC, UTFVI values were divided into 6 classes (i.e., none, weak, medium, strong, stronger, and strongest), according to the well-consolidated thresholds derived in [24,40] and reported in Table 2. Moreover, the table reports the correspondence of each UTFVI class with the class of the ecological evaluation index.

#### 2.2.3. LULC Processing

The LULC processing was performed starting from Landsat Level-2 collections as specified in Table 3. The Landsat imagery obtained is classified into four LULC classes, namely vegetation, built-up, bare-land, and water, using the support vector machine (SVM) classifier in ArcGIS Pro 3.1.0. Firstly, the classification schema were developed, and training samples were created. Then, these were fed to the SVM algorithm to classify the imagery. The SVM algorithm operates by identifying the hyperplane in a dataset that most effectively divides two classes. The hyperplane is selected to optimize the distance between it and the nearest data points in each class [41]. The classification was followed by the accuracy assessment of the classified datasets. A total of 506 to 508 samples were automatically generated by ArcGIS Pro 3.1.0 using the stratified random sampling technique [42,43]. The comparison produced the confusion matrix, which led to accuracy and kappa index calculations [32]. This was performed manually by comparing the classified maps with the input satellite imagery for each of the chosen samples.

#### 2.2.4. Prediction of LULC and UTFVI Change for the Year 2030

The cellular automata model was utilized to simulate the potential future change of LULC classes. This simulation aimed to predict the likelihood of changes in land use by employing an artificial neural network (ANN) learning process. The prediction process involved the utilization of the Modules for Land Use Change Evaluation (MOLUSCE) plugin [44]. QGIS version 2.18 was used as the software for this analysis. The CA-ANN algorithm was trained using the maps of 1990 and 2000 to predict the LULC map for the year 2010. The supplementary spatial variables, such as the digital elevation model (DEM), primary and secondary school buildings, railway stations slope map, rivers, roads and railway tracks were included. LULC maps corresponding to years 2000 and 2010 were used to predict the LULC scenario for the year 2022. The predicted 2010 and 2022 LULC maps were then compared with the classified 2010 and 2022 maps to evaluate CA-ANN model accuracy. Moreover, LULC maps of the years 2010 and 2022 were used to predict the LULC scenario for the year 2030. The input variables used in predicting the LULC for the year 2030 were the LULC maps of 2010 and 2022. These predictions are based on the same spatial variables as before with the addition of the residential building records. To develop the transition potential model, 3000 samples were randomly selected using a random sampling technique. Considering the literature, the neighborhood rule was limited to three pixels, and the model’s learning rate was set to 0.100 [45]. The simulation consisted of 500 iterations to capture the shifting pattern for the year 2010. The procedure involved calculating the percentile area change and generating a transition matrix that represents the ratio of moving pixels within the LULC classes. A LULCC map, for the period between 1990 and 2000, was generated. Subsequently, a simulated LULC map for the year 2010 was predicted using an ANN multi-layer perceptron model [24]. To examine the relationship between spatial variables in two images, the Pearson’s correlation coefficient [46] was employed. The simulated LULC maps for the years 2010 and 2022 were validated against the reference LULC maps obtained through satellite image classification, exploiting the TerrSet 2020 software validation module. The validation process generates various kappa parameters, which serves as a measure of agreement between the simulated and reference maps. According to [32], a kappa parameter ranging from 0.0 to 0.20 indicates slight agreement, 0.21 to 0.40 suggests fair agreement, 0.41 to 0.60 reflects moderate agreement, 0.61 to 0.80 indicates substantial agreement, and a value greater than 0.80 signifies almost perfect agreement. Similarly, the simulated 2030 LULC map was obtained using the same model and feeding the 2010 and 2022 LULC maps as input maps. Furthermore, it is worth noting that the ROI contains natural parks and protected areas, where several activities are prohibited, including the construction of new buildings. Hence, to ensure compliance with regulations and to accurately reflect the restrictions on development, the prediction model was further optimized by excluding those areas. To predict the UTFVI for the year 2030, also other spatial variables, such as the DEM, distribution patterns of LST, and land cover indices like NDVI, the Normalized Difference Built-up Index (NDBI), and the Modified Normalized Difference Water Index (MNDWI), were calculated at about 10-year intervals (1990, 2000, 2010, and 2022). The CA-ANN model considers the transition from 2010 to 2022 to predict probabilities of future LULC changes for the year 2030.

## 3. Results and Discussion

### 3.1. LULC Scenario

**Figure 3:**
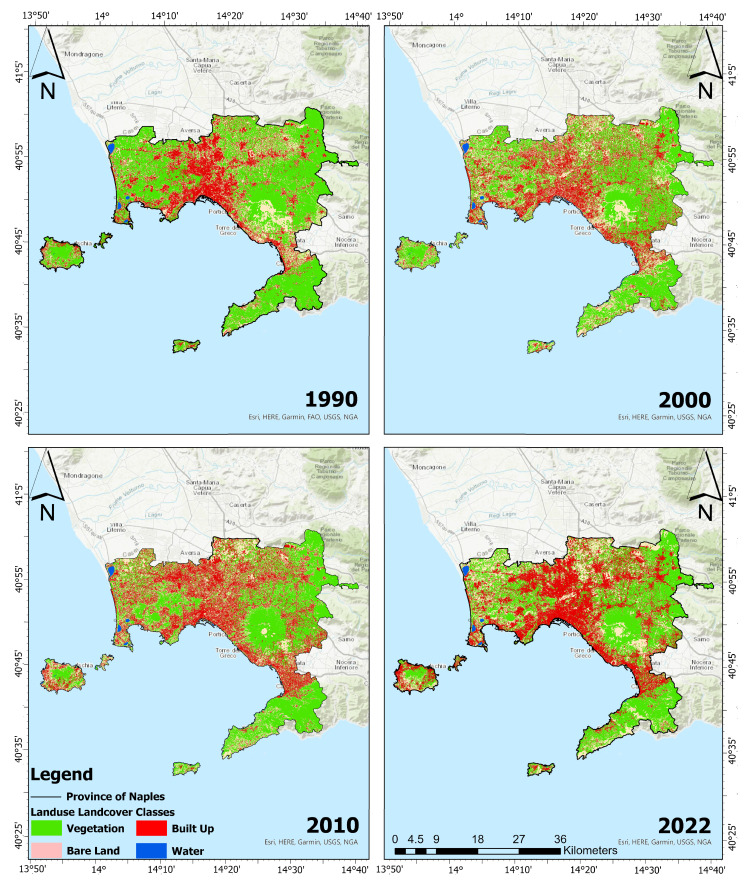
LULC scenario of the ROI. (**Top-left panel**) and (**top-right panel**) refer to 1990 and 2000, respectively. (**Bottom-left panel**) and (**bottom-right panel**) to 2010 and 2022, respectively. Green refers to vegetation class. Red refers to built-up class. Beige refers to bare-land, while blue to water class.

**Figure 4:**
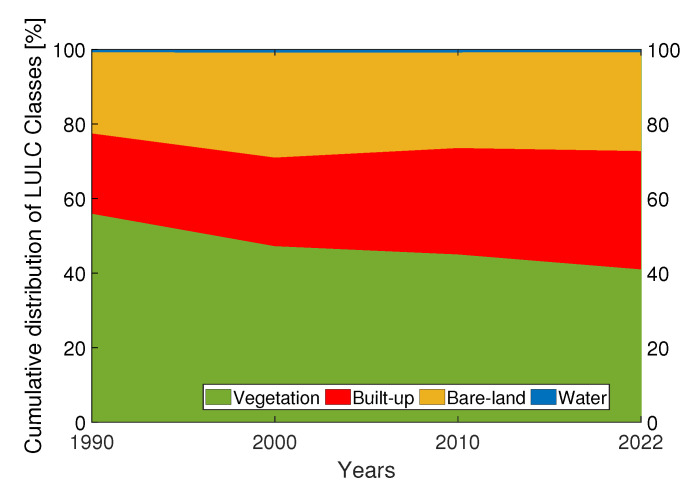
Changes in LULC classes from 1990 to 2022. On the x-axis are represented the four years analyzed (1990, 2000, 2010, and 2022), whereas the y-axis shows the corresponding cumulative distribution of LULC classes, in percentage. Green refers to vegetation class. Red refers to built-up class. Yellow refers to bare-land class, while blue to water class.

Figure 3 shows the estimated LULC maps from 1990 to 2022 for the four years considered (1990, 2000, 2010, and 2022). Figure 4 reports the changes in LULC classes from 1990 to 2022. Referring to Figure 3 and Figure 4, it can be noticed that, over the past 32 years, two notable trends have emerged in the shifting pattern of LULC. Firstly, there has been a significant increase in the built-up class (+10.21%), accompanied by a substantial decline in the vegetation class (−14.90%). Additionally, there has been a noticeable rise in bare-land (+4.70%). To evaluate the accuracy of the classified maps in representing the real world, an accuracy assessment was conducted, the results of which are presented in Table 4. The latter represents the confusion matrices for the years 1990, 2000, 2010, and 2022. Each value contained in the columns from the 3rd to the 6th represents the random sampled point. In particular, all bold values on the principal diagonals are correctly classified, whereas off-diagonal values are incorrectly classified. The last column of Table 4 reports the kappa index for each year analyzed. The classified maps for the years 1990, 2000, 2010, and 2022 exhibit an overall accuracy of at least 91% and a kappa index of at least 0.84, which indicates almost perfect agreement.

### 3.2. UTFVI Scenario

**Figure 5:**
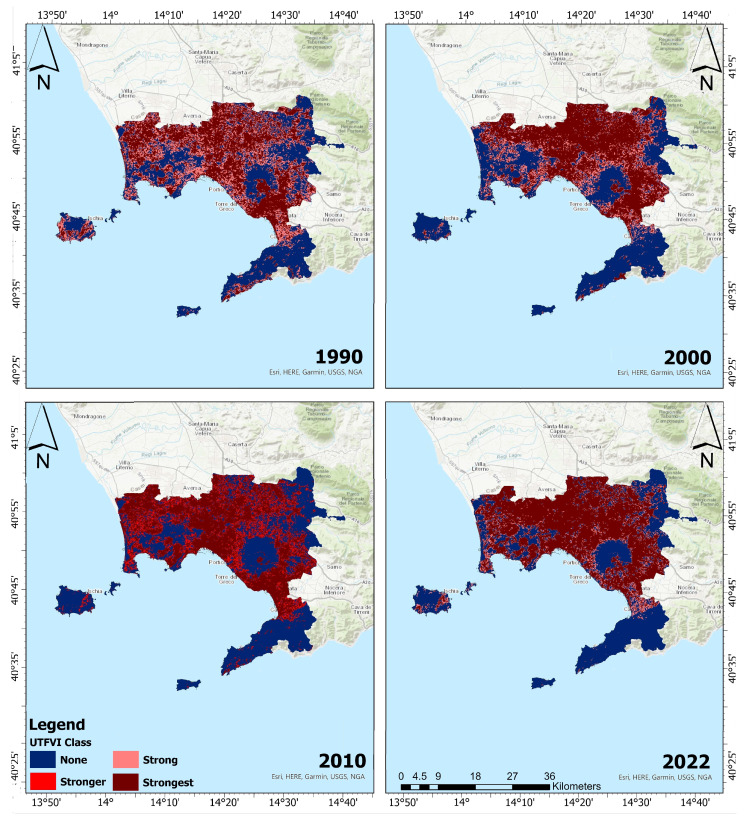
Summer spatial distribution of UTFVI zones for 1990 (**top-left panel**), 2000 (**top-right panel**), 2010 (**bottom-left panel**), and 2022 (**bottom-right panel**). Dark blue refers to none UTFVI areas, pink color to strong UTFVI areas, orange to stronger UTFVI areas, and dark red to strongest UTFVI areas.

**Figure 6:**
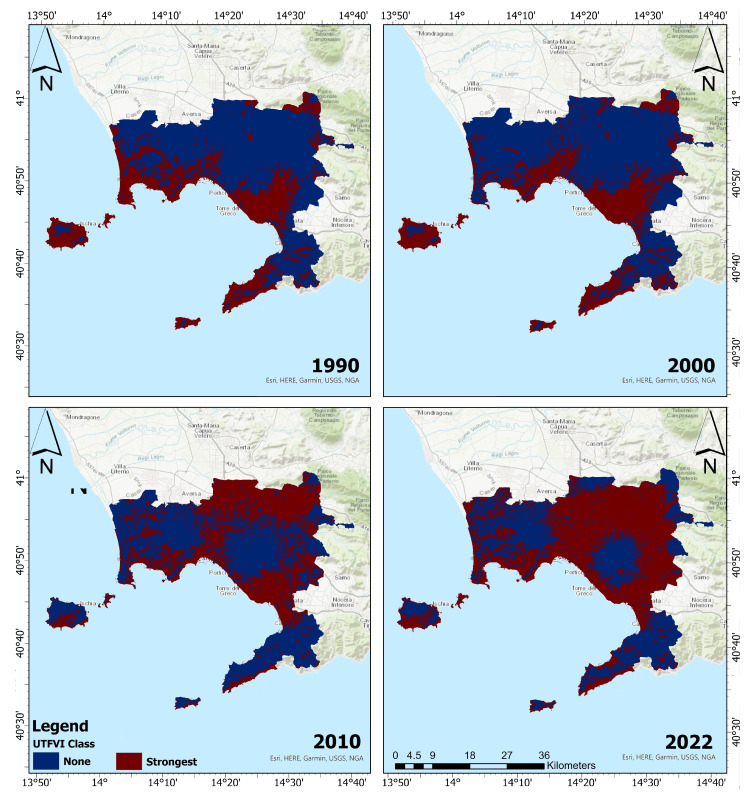
Winter spatial distribution of UTFVI zones for 1990 (**top-left panel**), (**top-right panel**), 2010 (**bottom-left panel**), and 2022 (**bottom-right panel**). Dark blue refers to none UTFVI areas, while dark red color to strongest UTFVI areas.

Figure 5 and Figure 6 depict the spatial distribution of UTFVI zones for summers and winters, respectively. It is worth noting that comparable patterns of variance in the extreme (none and strongest) categories of thermal assessment were seen in the ROI during the summer and winter seasons. Particularly, the worst UTFVI category migrated from the south towards northern and eastern parts of the ROI between 1990 and 2022.

The spatial distribution of seasonal UTFVI is presented in Table 5. In summer 1990, the areas with strong UTFVI were equal to 269.90 km${}^{2}$; they declined, in the summer of 2000, to 175.19 km${}^{2}$. It further declined in 2022 to 111.14 km${}^{2}$. Conversely, the regions with the strongest UTFVI exhibited a substantial upward trend throughout the study period. By 2022, over half of the research area contained regions with strongest UTFVI class, covering an area of 606.98 km${}^{2}$. Similarly, during the winter seasons, significant increases in UTFVI were observed between 1990 and 2022. The reduction in areas with the none UTFVI class was particularly noticeable during this season. The areas with none UTFVI experienced a decrease of 277.45 km${}^{2}$ over the period 1990–2022. In the winter of 2022, the strongest UTFVI covered an area of 676.59 km${}^{2}$. The distribution of seasonal UTFVI reveals that strongest UTFVI regions have significantly expanded, while none UTFVI zones have drastically decreased over time, throughout the ROI.

### 3.3. Distribution of UTFVI over Different LULC Classes

**Figure 7:**
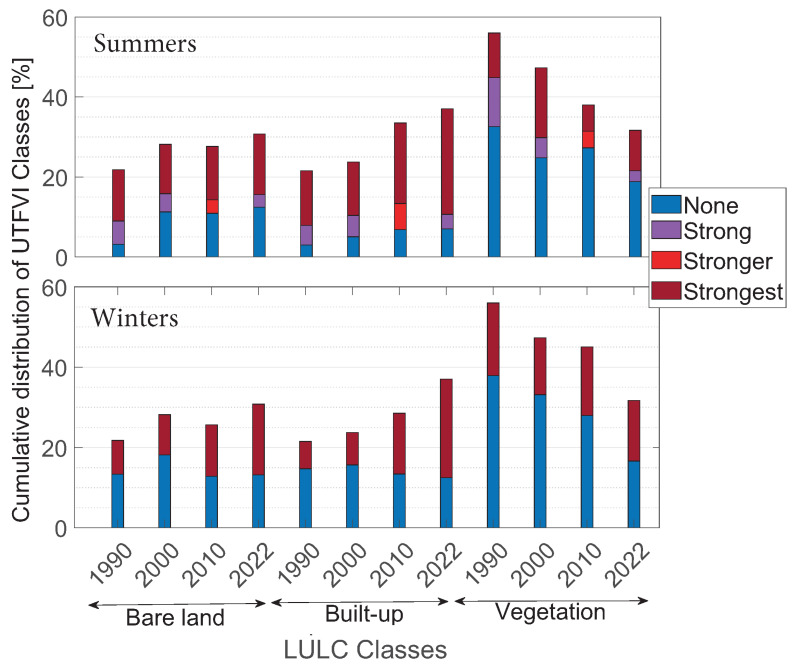
Cumulative distribution of UTFVI over LULC classes: (**top panel**) refers to summer seasons while (**bottom panel**) to winter seasons. On the x-axis are indicated the four LULC categories considered (bare-land, built-up, and vegetation) for each of the years considered (1990, 2000, 2010, and 2022). On the y-axis is represented the corresponding cumulative distribution of temperature zones, in percentage. Dark blue color refers to none UTFVI zone. Strong UTFVI class is represented in magenta, while stronger and strongest UTFVI classes are represented in red and dark red colors, respectively.

Summer and winter distributions of UTFVI classes over LULC categories are shown in Figure 7, in the top and the bottom panels, respectively. These values were obtained by means of the cross-tabulation tool in ArcGIS Pro 3.1.2.

In Figure 7 is shown a comparison between the LULC classes and the corresponding UTFVI for the different years analyzed. Each bar has different colors according to the UTFVI. The LULC class for water is not represented in the figure since it can be neglected, given its low areas. The top panel of Figure 7 shows summer results: the UTFVI effect ranges from none to strongest. The analysis reveals that the intensification of UTFVI was predominantly observed in the built-up class. The bottom panel of Figure 7 shows winter results: the UTFVI effect has only none and strongest UTFVI. Built-up and bare-land LULC classes were the only areas where UTFVI amplified.

### 3.4. Predicted Scenario of LULC and UTFVI

The ANN model used for LULC classification was validated for the year 2022. Additionally, the UTFVI model was validated for the year 2022 by comparing predicted and reference UTFVI maps. The validation parameters are listed in Table 6. The validation produced different kappa parameters: $K_{std}$ kappa for standard, $K_{no}$ kappa for no information, $K_{loc}$ kappa for location, and $K_{locstrata}$ kappa for stratum level location. $K_{std}$ computes the ratio of inaccurate allocations by chance to the correct assignments. $K_{no}$ represents the overall agreement between the predicted and reference map. $K_{loc}$ computes the spatial accuracy in the overall landscape by utilizing correct assignment values in each class between the predicted and reference map. The quantification of the spatial accuracy within pre-identified strata is given by $K_{locstrata}$, and it measures how well the grid cells are located within the strata. The average kappa parameters for the predicted LULC maps of 2010 and 2022 stand at 0.9041 and 0.8386, respectively, while the average kappa parameters for summer and winter predicted UTFVI are equal to 0.9622 and 0.8848, respectively. Furthermore, the agreement/disagreement component validation analysis, reported in Table 7, evaluates the deviations between the predicted and actual maps.

**Figure 8:**
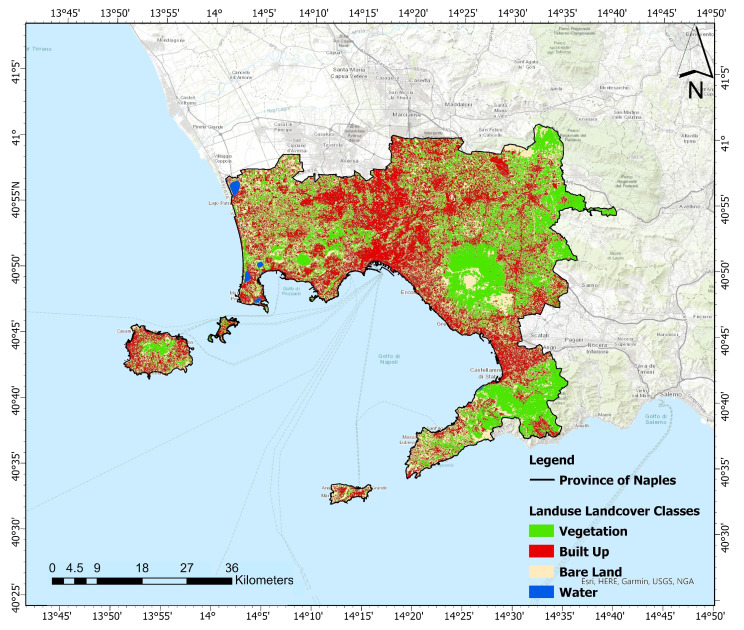
Predicted LULC scenario of the Province of Naples for the year 2030. Green color refers to vegetation class. Red color refers to built-up class. Beige color refers to bare-land, while blue color refers to water class.

The predicted LULC map for year 2030 is presented in Figure 8. In anticipation of urbanization and socioeconomic growth, it is expected that the built-up region will expand towards peri-urban areas. The growth of built-up areas is predicted to be substantial comparable to previous years, with other LULC classes undergoing transformation into built-up covers. By 2030, the built-up area is predicted to encompass 37.1% of the total provincial area. Concurrently, vegetation is forecast to decrease from 41.1% to 31.7% by 2030. Over the period from 1990 to 2030, the overall built-up area is predicted to grow by 15.5%. In contrast, vegetation is expected to decrease by 14.9%. Notably, also bare-land will undergo a transformation, experiencing an overall increase of 9.0% between 1990 and 2030.

**Figure 9:**
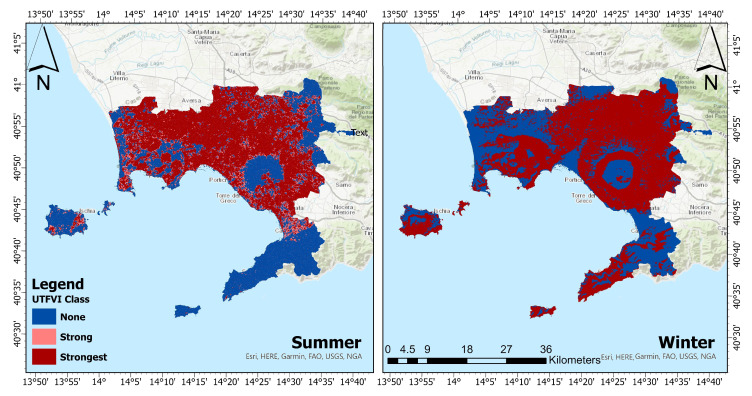
(**Left panel**): predicted UTFVI scenario of the Province of Naples for summer 2030. (**Right panel**): predicted UTFVI scenario of the Province of Naples for winter 2030. The blue, beige, and brown colors in top panels refer to none, strong, and strongest UTFVI, respectively.

Left panel of Figure 9 illustrates the predicted summer UTFVI map for 2030, indicating a shift from none UTFVI towards strong and strongest UTFVI. In the summers of 2030, the dominant effect will still be the strongest UTFVI, which is expected to remain quite stable, slightly increasing from 606.97 km${}^{2}$ to 616.22 km${}^{2}$, between 2022 and 2030. Simultaneously, the none and strong UTFVI areas are predicted to remain quite stable. The predicted winter UTFVI distribution for 2030 is depicted in the right panel of Figure 9, revealing a significant reduction in the thermal comfort zones of the ROI. The strongest UTFVI effect is expected to be predominant, increasing from 676.59 km${}^{2}$ to 731 km${}^{2}$, between 2022 and 2030. Conversely, the none UTFVI impacted areas will decrease from 497.41 km${}^{2}$ to 443 km${}^{2}$. A noticeable outward expansion in the built-up area is observed in almost every direction. In particular, the central northern regions have a higher growth rate of built-up areas as compared to other parts of the province, mainly at the expense of vegetation cover. A greater transition to bare-land was also observed over Vesuvius between the years 2010 and 2022. This trend could also be attributed to the huge forest fire event that occurred in 2017. Furthermore, a comparison between LULC maps of 1990, 2000, 2010, and 2022 revealed a steady transition of vegetation cover to bare-land in the Sorrento peninsula. The CA-ANN model further predicted amplifications in the built-up and bare-land classes, while significant decreases were predicted for the vegetation class. The model showed that the built-up areas will cover an area of 435 km${}^{2}$, with an increase of 5.3 percent as compared to 2022. The bare-land is predicted to cover an area of about 361 km${}^{2}$, registering an increase of 4.3 percent with respect to 2022. The vegetation class will further decline to 372 km${}^{2}$, registering a decrease of about 9.4 percent with respect to 2022. The prediction results demand necessary steps to be taken to preserve the diminishing of vegetation cover to maintain the ecological balance in the Province of Naples. The increase in built-up and bare-land areas could lead to the formation of more UHI, thus further worsening the thermal characteristics of the province. Another consequence resulting from the loss of green spaces can be the imbalance of the biodiversity in the province. Moreover, increased built-up and bare-land will reduce the ability to absorb the rainfalls, hence amplifying the susceptibility to floods. Thus, it can be stated that the preservation of natural resources is crucial; hence, the provincial authorities must guarantee that urban areas are developed according to a plan that does not compromise on diminishing green and water cover. The increase in built-up and bare-land experienced between 1990 and 2022 resulted in alterations in thermal attributes. It is worth noting that, in this period, the strongest UTFVI class expanded in all directions, particularly towards the northern and eastern regions of the province. This reflects in a more prominent worst class of the ecological evaluation index. The trend of the UTFVI classes is confirmed by the prediction of 2030. This will have significant impacts on the quality of life in the study area. In particular, if policies aimed at contrasting soil consumption (the so-called overbuilding) are not undertaken, we will face the worsening of the habitability of the area. A transition towards renewable energy sources instead of fossil fuels, the development of urban green spaces, and water-sensitive design practices in urban planning could be some ways to reduce the thermal effects.

## 4. Conclusions

The analysis of thermal characteristics offers valuable insights into the thermal patterns observed in the Province of Naples. By systematically analyzing satellite images, a comprehensive understanding of the thermal environment was achieved. Particularly, reflective and thermal bands from Landsat 4-5 TM and Landsat 9 OLI were employed. The findings of this study revealed a concerning trend of outward expansion in the built-up area of the Province of Naples, with central northern regions experiencing the highest growth rate, predominantly at the expense of vegetation cover. The Vesuvius area saw a notable transition to bare-land, possibly due to a significant forest fire event in 2017. Over the decades, the steady conversion of vegetation cover to bare-land in the Sorrento peninsula is evident. The CA-ANN model predictions highlight the amplification in built-up and bare-land areas and a significant decrease in vegetation cover by 2030. The expansion of built-up and bare-land areas between 1990 and 2022 led to notable changes in thermal attributes. During this period, the strongest UTFVI class expanded in all directions, especially towards the northern and eastern regions of the province, resulting in a more pronounced worst class in the ecological evaluation index. The prediction for 2030 confirms this trend, which is anticipated to have significant impacts on the quality of life in the study area. Addressing soil consumption through appropriate policies becomes crucial to prevent further deterioration in habitability. These predictions emphasize the urgent need for proactive measures to preserve and protect the diminishing vegetation cover to maintain ecological balance, combat the urban heat island effect, and safeguard biodiversity in the province. Additionally, the increased built-up and bare-land areas could exacerbate the vulnerability to floods, necessitating careful urban planning to prioritize the preservation of natural resources. By promoting sustainable development practices, such as the adoption of renewable energy sources, the creation of urban green spaces, and water-sensitive design, the province can mitigate the adverse thermal effects and enhance the quality of life for its residents. It is crucial for provincial authorities to take decisive actions now to secure a sustainable and resilient future for the region. Our findings revealed that the vegetation cover will diminish; thus, it could be interesting to carry out in-depth research about the corresponding changes in the carbon sequestration in the same area. The investigation of the relationship between concentrations of free and trapped carbon and LULC dynamics can further assist the development of efficient strategies to mitigate these effects.

## Figures and Tables

**Table 1:** Description of the collected Landsat images for LST processing.

Season	Month	Year	Satellite Mission	Sensor	Bands (LST)
Summer	24 July	1990	Landsat 4	TM	3, 4, 6
	26 August	2000	Landsat 5	TM	3, 4, 6
	22 August	2010	Landsat 5	TM	3, 4, 6
	14 July	2022	Landsat 9	OLI	4, 5, 10
Winter	21 December	1990	Landsat 4	TM	3, 4, 6
	27 December	2000	Landsat 5	TM	3, 4, 6
	28 December	2010	Landsat 5	TM	3, 4, 6
	19 January	2022	Landsat 9	OLI	4, 5, 10

**Table 2:** The threshold of ecological evaluation index and UTFVI.

Threshold Value	UTFVI Class	Ecological Evaluation Index
<0	None	Excellent
0–0.005	Weak	Good
0.005–0.010	Middle	Normal
0.010–0.015	Strong	Bad
0.015–0.020	Stronger	Worse
>0.020	Strongest	Worst

**Table 3:** Description of the collected Landsat images for LULC processing.

Season	Month	Year	Satellite Mission	Sensor	Reflective Bands
Summer	24 July	1990	Landsat 4	TM	1–5&7
	26 August	2000	Landsat 5	TM	1–5&7
	22 August	2010	Landsat 5	TM	1–5&7
	14 July	2022	Landsat 9	OLI	1–7
Winter	21 December	1990	Landsat 4	TM	1–5&7
	27 December	2000	Landsat 5	TM	1–5&7
	28 December	2010	Landsat 5	TM	1–5&7
	19 January	2022	Landsat 9	OLI	1–7

**Table 4:** Confusion matrices for the years 1990, 2000, 2010, and 2022 and corresponding kappa in for the accuracy assessment of LULC classification.

Year	LULC Class	Vegetation	Built-up	Bare-land	Water	Total	User Accuracy
1990	Vegetation	**281**	5	1	0	287	0.98
	Built-up	7	**126**	0	0	133	0.95
	Bare-land	15	15	**46**	0	76	0.61
	Water	0	1	1	**9**	11	0.82
	Total	303	147	48	9	507	
	Producer Accuracy	0.93	0.86	0.96	1	**Overall Accuracy 91%**	**Kappa index 0.84**
2000	Vegetation	**154**	1	2	0	157	0.98
	Built-up	4	**214**	21	0	239	0.90
	Bare-land	6	5	**91**	0	102	0.89
	Water	0	0	0	**10**	10	1
	Total	164	220	114	10	508	
	Producer Accuracy	0.94	0.97	0.80	1	**Overall Accuracy 92%**	**Kappa index 0.88**
2010	Vegetation	**214**	3	8	0	225	0.95
	Built-up	9	**185**	13	0	207	0.89
	Bare-land	6	7	**51**	0	64	0.80
	Water	0	1	1	**8**	10	0.80
	Total	229	196	73	8	506	
	**Producer Accuracy**	0.93	0.94	0.70	1	**Overall Accuracy 91%**	**Kappa index 0.85**
2022	Vegetation	**154**	1	2	0	157	0.98
	Built-up	4	**214**	21	0	239	0.90
	Bare-land	6	5	**91**	0	102	0.89
	Water	0	0	0	**10**	10	1
	Total	164	220	114	10	508	
	**Producer Accuracy**	0.94	0.97	0.80	1	**Overall Accuracy 92%**	**Kappa index 0.88**

**Table 5:** Areas covered by different UTFVI classes (in percentage of the total).

Season	Year	None [%]	Weak [%]	Middle [%]	Strong [%]	Stronger [%]	Strongest [%]
Summer	1990	39.47	0.00	0.00	22.99	0.00	37.54
	2000	41.92	0.00	0.00	14.92	0.00	43.16
	2010	45.85	0.00	0.00	0.00	14.06	40.09
	2022	38.82	0.00	0.00	9.47	0.00	51.71
	2030	37.43	0.00	0.00	10.08	0.00	52.49
Winter	1990	66.00	0.00	0.00	0.00	0.00	34.00
	2000	67.43	0.00	0.00	0.00	0.00	32.57
	2010	54.81	0.00	0.00	0.00	0.00	45.19
	2022	42.36	0.00	0.00	0.00	0.00	57.64
	2030	37.73	0.00	0.00	0.00	0.00	62.27

**Table 6:** ANN model validation with Kappa parameter using validation module in TerrSet. The first two rows refer to summer and winter 2022 predicted maps, whereas third and last rows refer to predicted LULC maps for years 2010 and 2022, respectively.

Type	Year	$K_{\mathrm{Std}}$	$K_{\mathrm{no}}$	$K_{\mathrm{loc}}$	$K_{\mathrm{locstrata}}$	$K_{\mathrm{avg}}$
UTFVI summer	2022	0.9311	0.9536	0.9822	0.9822	0.9622
UTFVI winter	2022	0.8365	0.8815	0.9107	0.9107	0.8848
LULC	2022	0.7668	0.8554	0.8662	0.8662	0.8386

**Table 7:** Results of validation analysis (agreement/disagreement components values) in TerrSet 2020.

Agreement/Disagreement	UTFVI Summer	UTFVI Winter	LULC 2022
Agreement due to chance	0.2500	0.3333	0.2000
Agreement due to quantity	0.2441	0.1833	0.3039
Agreement at stratum level	0.0000	0.0000	0.0000
Agreement at gridcell level	0.4710	0.4044	0.3804
Disagreement at gridcell level	0.0085	0.0396	0.0588
Disagreement at stratum level	0.0000	0.0000	0.0000
Disagreement due to quantity	0.0263	0.0394	0.0569

## Data Availability

All relevant data are included in the manuscript.

## Abbreviations

The following abbreviations are used in this manuscript:

ANN	Artificial Neural Networks
BT	Brightness Temperature
CA	Cellular Automata
DEM	Digital Elevation Model
DN	Digital Number
GNSS	Global Navigation Satellite System
LST	Land Surface Temperature
LULC	Land Use Land Cover
LULCC	Land Use Land Cover Changes
MNDWI	Modified Normalized Difference Water Index
MOLUSCE	Modules for Land Use Change Evaluation
NDVI	Normalized Difference Vegetation Index
NDBI	Normalized Difference Built-up Index
NIR	Near Infrared
OLI	Operational Land Imager
ROI	Region of Interest
RTE	Radiative Transfer Equation
SUHI	Surface Urban Heat Island
SVM	Support Vector Machine
TIR	Thermal Infrared
TIRS	Thermal Infrared Sensor
TM	Thematic Mapper
TOA	Top of Atmosphere
USGS	United States Geological Survey
UTFVI	Urban Thermal Field Variance Index
